# Pubic Bone Aplasia as an Incidental Finding in the Adult Population: Case Report and Review of the Literature

**DOI:** 10.7759/cureus.12703

**Published:** 2021-01-14

**Authors:** Vasileios K Mousafeiris, Konstantinos Nikolopoulos, Thomas Repantis

**Affiliations:** 1 Orthopedics and Traumatology, General Hospital of Patras "Saint Andrews", Patras, GRC

**Keywords:** pubic aplasia, pubic bone aplasia, pubic rami aplasia, unilateral pubic aplasia, superior pubic rami, dysplastic acetabulum, undescended testes, hip dysplasia, pelvic dysplasia, pubic bone

## Abstract

Pubic bone aplasia is a rare finding that is either diagnosed as incidental finding or associated with various clinical syndromes. It is usually discovered in early childhood, however, there are few reported cases of late discovery during adulthood. We present a case of a 64-year-old male with unilateral superior pubic rami aplasia, discovered incidentally during workup for sustained trauma. Our patient reported treatment for unilateral hip dislocation in his early childhood and had a history of operated undescended testes ipsilaterally. This exact constellation of pubic rami aplasia, undescended testes and hip dysplasia is unique in the available literature. Even though our patient had a normal life and the pubic aplasia was discovered incidentally, it is important to always assess these patients for systemic involvement, either from the musculoskeletal system or other organs, in order to provide better treatment for them.

## Introduction

Pubic bone abnormality is relatively rare among the general population. Few cases have been published in the international literature and most of them have been congenital, presenting early in childhood. Some cases are associated with other anatomical or functional abnormalities or can occur within the spectrum of clinical syndromes. We present a case of unilateral superior pubic rami aplasia with associated congenital hip dysplasia in a 64-year-old male, that was discovered incidentally during workup for a lumbar spine fracture. Patient had also a history of operated undescended testis and reported treatment for hip dislocation ipsilaterally during childhood. To the best of our knowledge, this exact constellation of symptoms has not been presented before.

## Case presentation

**Figure 1:**
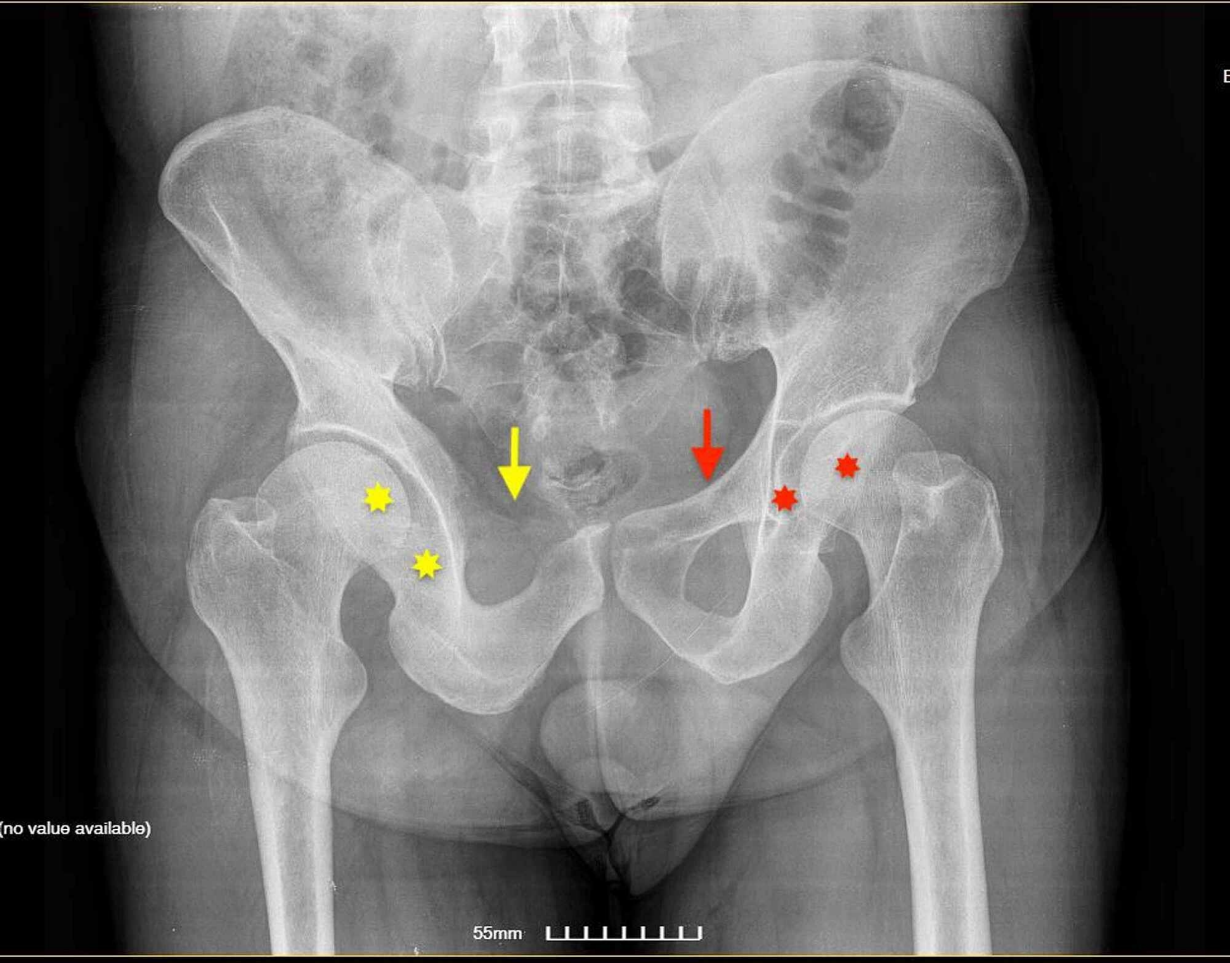
Anteroposterior (AP) X-ray of the pelvis reveals absence of the right superior pubic rami (yellow arrow). Note the normal left superior pubic rami (red arrow). Note, also, the dysplastic hip and dysplastic acetabulum ipsilaterally (yellow stars) and concern for dysplastic hip and acetabulum on the contralateral side (red stars). Initial imaging did not reveal abnormality of the femur shaft (up to its middle part, that was depicted).

A 64-year-old male presented to our emergency department after a fall from 3 meters height and landing on his feet. After medical clearance from the ER trauma team on primary survey, he was evaluated as a secondary survey by the orthopedic surgeons. Patient was mainly complaining of pain in the lumbar area. Inspection revealed no obvious abnormality of the spine, pelvis or hips, no open wound, no hematoma, no erythema, no swelling, no atrophy or hypertrophy of the body. Palpation revealed tenderness along the vertebrae of the lumbar spine. No other tender area at the back noted. No tenderness noted on the examination of the pelvis, both hips or any other parts of the body. The open book test for pelvic fracture or instability was negative. Both passive and active range of movement (ROM) for both hips was within normal range. Both lower limbs were neurovascularly intact; reflexes were normal bilaterally. Among others, X-rays of the pelvis, cervical, thoracic and lumbar spine were performed; they revealed fracture of the anterior column of the third lumbar vertebra. The fracture was stable, non-comminuted, non-displaced and the patient was treated conservatively with lumbar spine brace. Furthermore, X-ray of the pelvis revealed abnormal image of the right superior pubic rami, dysplastic hip and dysplastic acetabulum ipsilaterally and concern for dysplastic hip and acetabulum on the contralateral side. The initial imaging did not reveal abnormality of the femur shaft (up to its middle part, that was depicted) (Figure 1). Although the patient did not complain of pain or tenderness on the pelvis or the right hip region and had no previous history of trauma or pain regarding the above areas, we were concerned by this “osteolytic like'' image of the right superior rami. However, the patient did not recall having any older pelvis or hip X-rays to compare them with our current ones.

**Figure 2:**
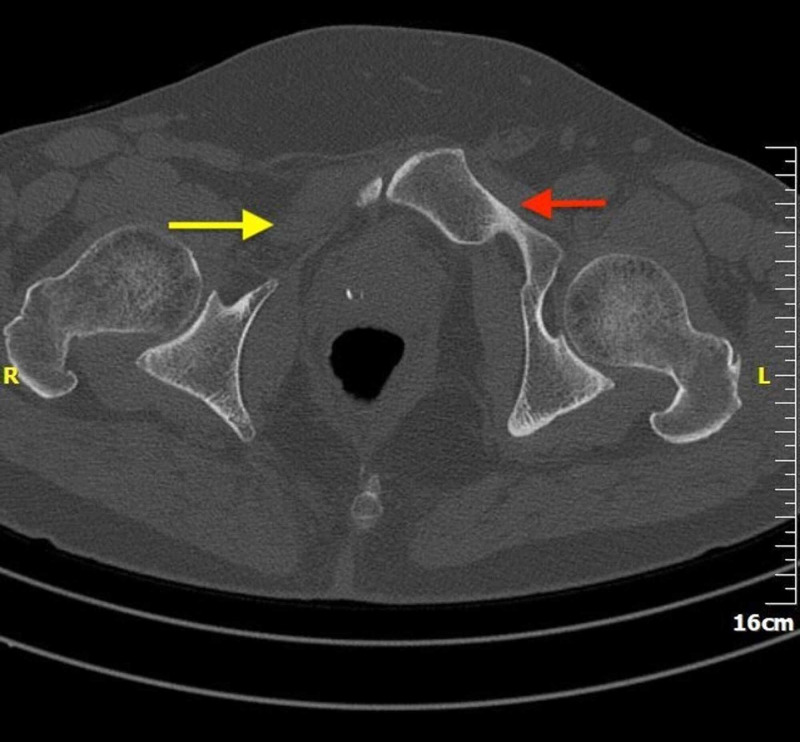
CT scan of the pelvis (axial view): absence of the left superior pubic rami (yellow arrow). Note the normal right superior pubic rami (red arrow).

**Figure 3:**
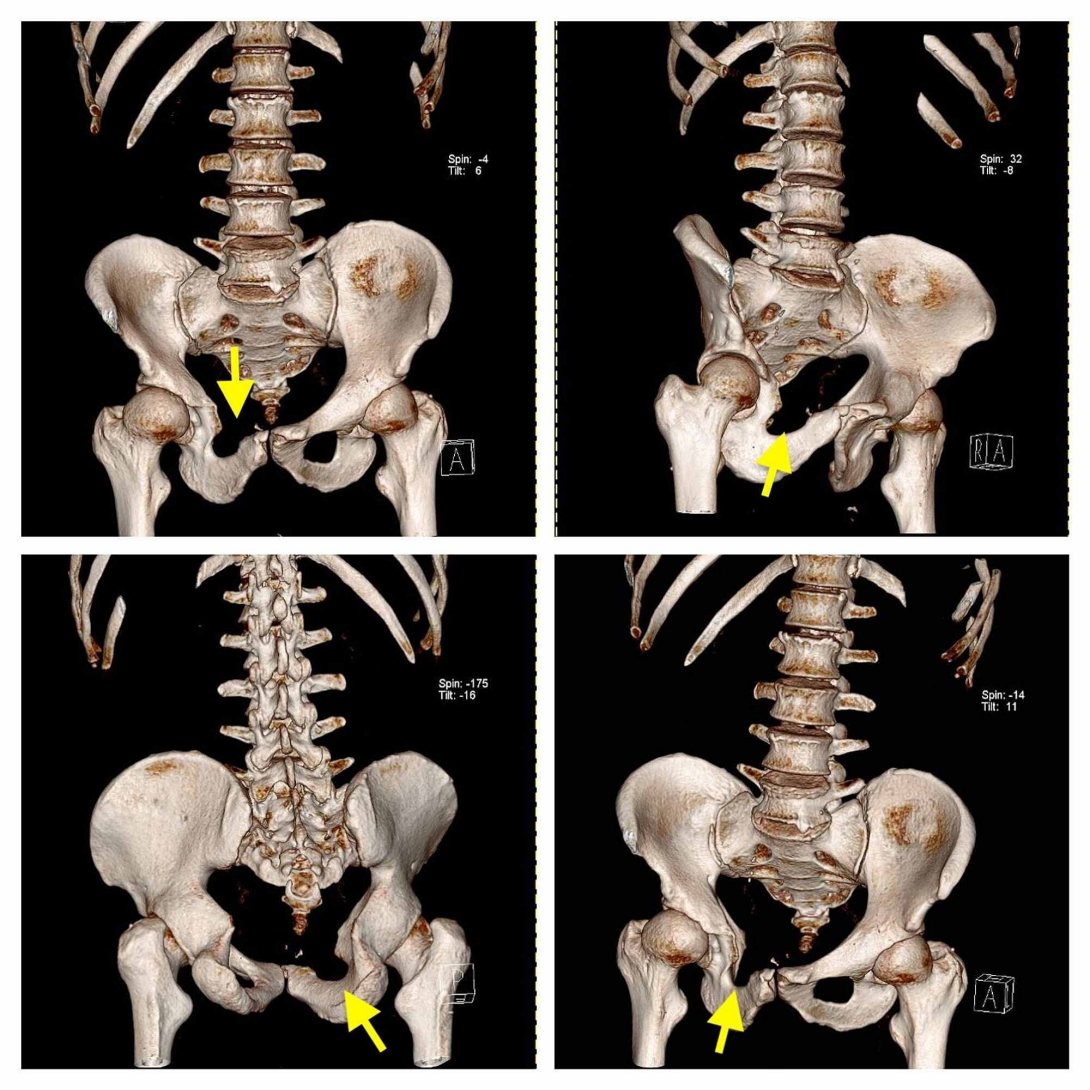
CT 3D reconstruction of the pelvis which shows the absence of the right superior pubic rami (yellow arrows).

To further evaluate the extent of the bony defect of the pubic rami, the patient was given an appointment with our outpatient clinic department. History of present illness revealed congenital dysplastic hip with a reported treatment for hip dislocation at around four years of age. The patient could recall having a Pavlik harness for a few months, though exact timing and details were unclear as there are no records of the procedures and the patient reproduced only the information that he was told from his parents. Patient was also complaining about mild ipsilateral hip instability, though it was not debilitating and the patient was coping without issues. Hip instability did not affect his life, to the extent that he has been working full time as construction worker. Patient had also undergone an operation for undescended right testis (ipsilateral with the pubic bone abnormality) at the age of 10. Patient did not recall having problem with urination or history of genitourinary anomalies. Patient also did not complain of shoulder or knee pain. He has no family history of hip dislocations, pelvic ossification abnormalities or genitourinary abnormalities up to his third-degree relatives (parents, children and siblings). Thorough physical exam was negative for other deformities or abnormalities of any part of the body. A CT scan of the pelvis and hips was subsequently performed. CT imaging revealed aplasia of the right superior pubic rami, confirmed the dysplastic hip and acetabulum ipsilaterally and mildly dysplastic hip on the opposite side and excluded tumor-like lesions (Figure 2). A 3D reconstruction of the pelvis was also performed, which confirmed the findings (Figure 3).

The patient was, also, referred to the Urology department for further testing, which did not reveal any GU abnormality. He was, therefore, subsequently, followed in our outpatient department for the lumbar vertebra fracture, which was his main complaint and not associated with the pubic rami aplasia. 

## Discussion

Recognition of aplasia of pubic bone is of clinical importance because it is sometimes associated with clinical syndromes and seen in combination with other abnormalities of the body. When first seen in adulthood it could also be misdiagnosed as an osteolytic lesion. In a few cases, it has been reported as isolated lesion. In our case, a 64-year-old male was diagnosed with pubic aplasia of the superior pubic rami discovered incidentally during workup for lumbar fracture, along with dysplastic acetabulum ipsilaterally and dysplastic hips bilaterally, as well as operated undescended testes on the same side of the pubic bone aplasia. To the best of our knowledge, this exact constellation of symptoms has not been presented in the literature before. It is critical to always search for concomitant abnormalities that co-exist with pubic aplasia in order to provide better care and improve the outcome of our patients. 

The development of the pelvic bone is not well investigated. Malaschicev et al. reported that pubic and iliac bones develop differently from the iliac bone [1]. Ischiopubic bones start developing antenatally at around the fifth or sixth month of fetal life [1]. At birth, pubic bone is usually ossified; however, the symphysis pubis is ossified during puberty and adolescence [2,3]. Incomplete ossification of the pubic bone has been described either in endocrine conditions or associated with various clinical syndromes [4]. 

Few cases similar to ours have been reported in the literature. Sarban et al. presented a very similar case of one-year-old boy with teratogenic hip dislocation, absence of the ipsilateral pubic bone, undescended testis ipsilaterally and hypospadias [5]. Yildiz et al. reported a case of a two-year-old patient with bilateral agenesis of pubic bones along with bilateral undescended testes. [6]. These cases have clinical picture similar to our case, but the exact constellation of symptoms in our patient is, to the best of our knowledge, unique, especially in the adult population. Although Churchill et al. report that aplasia of the pubic bone does not predispose to hip dislocation, our patient had reported treatment for hip dislocation early in childhood [7]. He remained, thereafter, free from symptoms for almost 60 years. 

Further authors have described lesion of the pubic bone along with other abnormalities of the musculoskeletal tissue and beyond. Pubic bone aplasia is associated with genitourinary (GU) abnormalities. It is associated with epispadias and bladder extrophy [7]. Sponseller et al., in particular, analyzed 24 CT scans of patients with bladder exstrophy in comparison to age-matched controls and found that in the group with bladder exstrophy the incidence of pubic bone lesion was higher [8]. 

Pubic rami lesions are also associated with patella anomalies [9,10]. Nail-patella syndrome is an autosomal dominant inherited syndrome that usually runs in families. It presents with nail abnormalities and patella anomalies, but it can also present with anomalies of the elbow, as well as pubis and renal involvement [9]. Small patella syndrome is associated with absence of patella bones along with anomalies of the pubic and ischial bones [11]. Similarly, Habboub et al. [4] presented a case of an 11-year-old girl with bilateral absence of patella and ischiopubic bones along with other musculoskeletal anomalies. Our patient did not have clinical or radiological involvement of the patella, therefore any of the above syndromes are excluded.

Further rare constellations of lesions have been described. Genitopatellar syndrome consists of patellar lesions, other lower limb anomalies as well as genitourinary, CNS and facial involvement [12]. Sferopoulos et al. described four cases of ischiopubic hypoplasia; one along with scoliosis, another along with scoliosis and bilateral patella aplasia and two more children with hip dislocation [13]. 

Furthermore, pubic rami anomalies have been reported as part of more severe and complex clinical syndromes, such as cleidocranial dysostosis, metatropic dwarfism, spondyloepiphyseal dysplasia [4] and prune belly syndrome (PBS) [14]. Although our patient had ipsilateral undescended testis and musculoskeletal involvement, he lacked the obstructive uropathy and abdominal muscle anomaly of the PBS syndrome. Megarbane et al. presented a case of ischiopubic aplasia along with hip dislocation, short femurs and dysplastic tibia bilaterally [15]. 

On the other hand, isolated loss of pubic rami, although rare, has been reported. Schey et al. presented a family where the male members; father and three sons had various degrees of anomalous pubis ossification as isolated lesions [16]. Saber presented a case of a 35-year-old female with isolated loss of pubic rami [17]. Wahlquist et al. reported an 11-year-old girl complaining of intoeing with isolated unilateral superior pubic ramus aplasia [18]. Common denominator to those cases is that they presented for a reason different than pelvic involvement, they had normal lives and the pubic bone anomaly was discovered accidentally. Our case can be considered similar to those cases, because our patient had a normal life after the initial treatment of the hip dislocation in his early childhood and the pubic bone aplasia was discovered accidentally late in his adult life. 

## Conclusions

Pubic bone dysplasia can occur in association with clinical syndromes or present as isolated lesion. Although it is usually noticed as part of the workup in early childhood, there have been cases that the pubic bone abnormality is discovered in adulthood and the patients had almost normal life. It is important to investigate further any case of pubic bone abnormality, in order to exclude other pathologies or involvement of other systems and provide better care for our patients.
